# Supplementary material for: Structural dynamics of single SARS-CoV-2 pseudoknot molecules reveal topologically distinct conformers
Source: Nat Commun. 2021 Aug 6;12:4749. doi: 10.1038/s41467-021-25085-6 (PMC8346527; doi:10.1038/s41467-021-25085-6)
Supplement: Supplementary file 1 — Supplementary Information [file 41467_2021_25085_MOESM1_ESM.pdf]

Supplementary Material for:

Structural dynamics of single SARS-CoV-2 pseudoknot molecules reveal topologically distinct conformers

Krishna Neupane[1*], Meng Zhao[1*], Aaron Lyons[1], Sneha Munshi[1], Sandaru M. Ileperuma[1], Dustin B. Ritchie[1], Noel Q. Hoffer[1], Abhishek Narayan[1], Michael T. Woodside[1,2]

[1]*Department of Physics, University of Alberta, Edmonton AB T6G 2E1, Canada*
[2]*Li Ka Shing Institute of Virology, University of Alberta, Edmonton AB T6G 2E1, Canada*

[*]These authors contributed equally

Corresponding author: Michael Woodside
Email: michael.woodside@ualberta.ca

This PDF file includes:

Supplementary Tables 1–5
Supplementary Figures 1–5

Supplementary Table 1: Length changes for unfolding and refolding transitions. State names are shown in FECs in Figs. 2–4 and Supplementary Fig. 4. Subscript “r” indicates results from refolding. Expected values are based on the structural models shown in Supplementary Fig. 4. Errors on measured values represent standard error on the mean.

State name	Observed ΔLc (nm)	Proposed structure	Expected ΔLc (nm)	Number of FECs	Number of molecules
Construct with 6 nt spacer between handle and 5′ end of stem 1, Mg^{2+}, no oligos					
N/N′	35.6 ± 0.4	Pseudoknot (threaded/unthreaded)	34.7-36.3[1]	3650	10
N/N'_r	35.3 ± 0.6	Pseudoknot (threaded/unthreaded)	34.7-36.3[1]		
I_1	17.5 ± 0.4	Stem 1 only	16.7		
I_{r1}	17.9 ± 0.6	Stem 1 only	16.7		
I_{r2}	29.2 ± 0.6	Stems 1 and 3	29.8		
Alt	25 ± 1	Alternate stem-loop	26.7		
Construct with 6 nt spacer between handle and 5′ end of stem 1, Mg^{2+}, oligo 1					
I_3	31 ± 1	Stems 2 and 3	30.3	180	2
I_4	8 ± 1	Lower part of stem 3	7.2		
Construct with 6 nt spacer between handle and 5′ end of stem 1, Mg^{2+}, oligo 2					
I_5	29.3 ± 0.6		29.8	910	3
I_1'	19.7 ± 0.7		19.0		
Construct with 6 nt spacer between handle and 5′ end of stem 1, no Mg^{2+}/oligos					
N″	34.3 ± 0.6	Pseudoknot (threaded/unthreaded)	35.4-36.1[2]	580	2
Construct with 1 nt spacer between handle and 5′ end of stem 1, Mg^{2+}, no oligos					
N/N′	35.1 ± 0.3	Pseudoknot (threaded/unthreaded)	34.7-36.3[1]	982	8
N/N'_r	34.9 ± 0.6	Pseudoknot (threaded/unthreaded)	34.7-36.3[1]		
I_1	17.5 ± 0.4	Stem 1 only	16.7		
I_{r1}	17.7 ± 0.6	Stem 1 only	16.7		
I_{r2}	28 ± 1	Stems 1 and 3	29.8		
Alt	25.0 ± 0.4	Alternate stem-loop	25.5[3]		
Construct with −2 nt spacer between handle and 5′ end of stem 1, Mg^{2+}, no oligos					
N_{-2}	33.2 ± 0.6	Pseudoknot (threaded/unthreaded)	34.6-35.3[4]	540	3
Alt_{-2}	22.5 ± 0.6	Alternate stem-loop	21.4[3]		

[1]Lengths vary depending on the structural model, because of differences between the locations of the termini.
[2]The expected ΔL_c without Mg^{2+} is based on simulations from Ref. 18, which may overestimate ΔL_c if interactions at the 5′ end are Mg^{2+}-dependent.
[3]The expected ΔL_c for the alternative stem loops should be shorter as the handle is extended toward stem 1.
[4]The effect of extending the handle into stem 1 is estimated from models in Refs. 15 and 18, but the estimates do not include possible distortions from the disruption of stem 1 and its stacking with stem 3.

Supplementary Table 2: Energy landscape fit parameters. Errors represent standard error on the mean from bootstrapping analysis.

Spacer length between handle and stem 1 (nt):	Lower-force population			Higher-force population		
	$\ln(k_0)$ [s^{-1}]	$\Delta x^‡$(nm)	$\Delta G^‡$(kJ/mol)	$\ln(k_0)$ [s^{-1}]	$\Delta x^‡$(nm)	$\Delta G^‡$(kJ/mol)
6	−5 ± 1	2.1 ± 0.3	33 ± 5	−4 ± 1	0.7 ± 0.1	31 ± 4
1	−6 ± 1	2.8 ± 0.6	33 ± 8	−3.5 ± 0.3	0.7 ± 0.1	40 ± 10
−2 (invades stem 1)	−2.8 ± 0.4	1.5 ± 0.2	27 ± 6	−5.4 ± 0.6	0.7 ± 0.1	29 ± 6
6 (without Mg^{2+})	−8 ± 1	4.0 ± 0.6	40 ± 6	−7 ± 1	2.8 ± 0.7	35 ± 6

Supplementary Table 3: Conformer proportions. Proportions of states N, N′, and Alt observed in unfolding FECs. Errors on Alt and N+N′ represent standard error of proportion; errors on N and N′ represent fitting error from two-component fits of force distributions.

Spacer length between handle and stem 1 (nt):	**N**	**N′**	**N + N′**	**Alt**
6	75 ± 2%	7 ± 2%	82 ± 1%	18 ± 1%
1	62 ± 2%	16 ± 2%	78 ± 2%	22 ± 2%
−2 (invades stem 1)	45 ± 3%	36 ± 3%	81 ± 3%	19 ± 3%
6 (without Mg^{2+})	39 ± 8%	58 ± 8%	97 ± 1%	3 ± 1%

Supplementary Table 4: RNA construct sequences. Pseudoknot sequence in black, single-stranded spacer sequence at 5′ end in red, single-stranded spacer sequence at 3′ end in blue. Underlined bases are annealed to DNA handle in the construct with −2-nt spacer.

Spacer length (nt)	**RNA sequence (5′ → 3′)**
−2	GCGGUGUAAGUGCAGCCCGUCUUACACCGUGCGGCACAGGCACUAGUAC UGAUGUCGUAUACAGGGCUU
1	UGCGGUGUAAGUGCAGCCCGUCUUACACCGUGCGGCACAGGCACUAGUA CUGAUGUCGUAUACAGGGCUU
6	GGGUUUGCGGUGUAAGUGCAGCCCGUCUUACACCGUGCGGCACAGGCA CUAGUACUGAUGUCGUAUACAGGGCUU
12	CUAUCAGGAACAGCGGUGUAAGUGCAGCCCGUCUUACACCGUGCGGCAC AGGCACUAGUACUGAUGUCGUAUACAGGGCUU

Supplementary Table 5: Primer and oligomer sequences. Oligomer sequences used as PCR primers to make handles, to modify pMluc-1 plasmid, and as anti-sense oligomers for blocking the folding of a specific parts of the pseudoknot.

**Primer/oligomer name**	**Sequence (5′ → 3′)**	**Plasmid/comments**
Construct design 1		
1,882-bp handle forward	TCTCACTCGGAAGGACATATGG	pMluc-1
1,882-bp handle reverse	GGATCCAGAGAAATGTTCTGG	
Transcription template (TT) forward	TAATACGACTCACTATAGGGTCTCACT CGGAAGGACATATGG	pMluc-1
TT reverse	TAGGAAACTTCTTAGCGCCCTC	
798-bp handle forward	ACTAGTCACGTGCCACCATG	pMluc-1
798-bp handle reverse	BioTEG-TAGGAAACTTCTTAGCGCCCT	
Construct design 2		
Oligo insert pMluc-1 forward	GATCCCGAATTCTGCAGCGGCCGCA GCTGGCGCGCCTCGAGC	Inserted into pMluc-1 between BamHI and XhoI sites to modify pMluc-1
Oligo insert pMluc-1 reverse	TCGAGCTCGAGGCGCGCCAGCTGCG GCCGCTGCAGAATTCGG	
2,075-bp handle forward	5DigN-GGAAGCTCCCTCGTGC GCTCTCCTG	pUC19
2,075-bp handle reverse	CGTCGT GACTGGGAAA ACCCTGGCG	
Oligo ligate forward	CCAGGGAGGCCCTTTCGTCGGGTAC	To ligate with PspGI-digested 2,075-bp handle to produce single stranded region at the 3'-end to anneal to 5'-end of RNA transcript
Oligo ligate reverse	CTCGAGGCGCGCCAGCTGCGGCCGC TGCAGAATTCGGTACCCGACGAAAGG GCCTC	
300-bp handle forward	ACTAGTCACGTGCCACCATG	pMluc-1
300-bp handle reverse	BioTEG-GAGCCGGTAGGAGCC ATTTC	
Transcription template (TT) forward	TAATACGACTCACTATAGGCGAATTCT GCAGCGGCCGCAG	Modified pMluc-1
TT reverse	GAGCCGGTAGGAGCCATTTCC	
Anti-sense oligomers		
Oligo 1	GCACTTACACCGC	Blocks folding of stem 1
Oligo 2	AGCCCTGTATACG	Blocks folding of stem 2

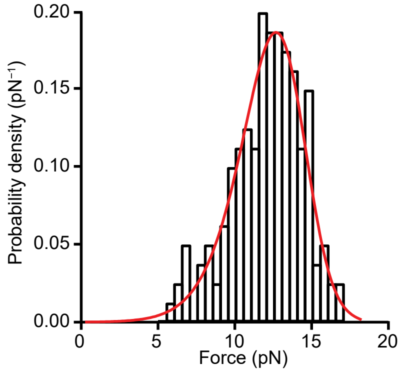

Supplementary Fig. 1: Unfolding force distribution for Alt. Fitting the distribution (black, N = 162 FECs from 8 molecules) to Eq. 2 (red) yields $\Delta x^{\ddagger}$= 4.5 ± 0.8 nm, indicative of secondary structure only.

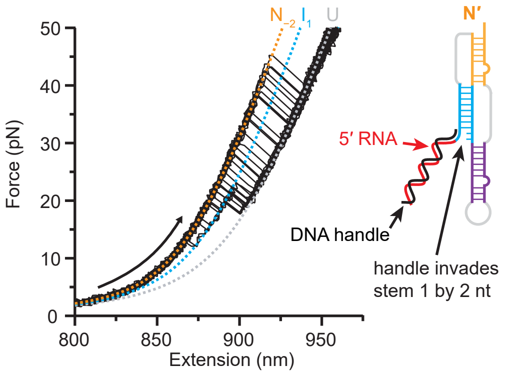

Supplementary Fig. 2: FECs measured with handle extending 2 nt into stem 1. Unfolding FECs show the same qualitative behavior seen in Fig. 2b and Fig. 3a, except that the total length change is ~2 nm shorter and there are significantly more unfolding events at lower force.

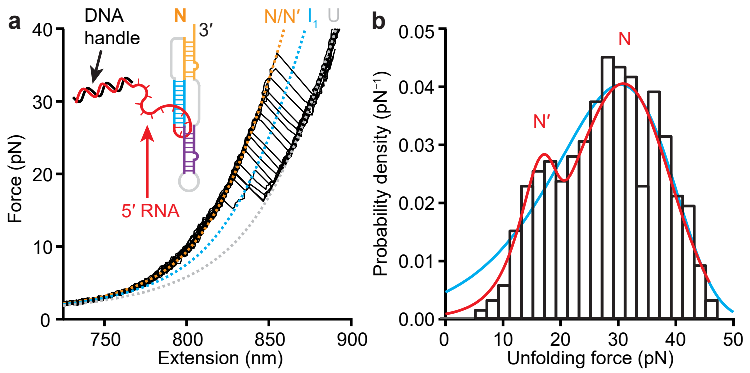

Supplementary Fig. 3: Increasing the spacer beyond 6 nt does not change the folding. (**a**) FECs measured on a construct with a 12-nt spacer between the DNA handle and the pseudoknot show the same behavior as seen in Fig. 2b. (**b**) Unfolding force distribution (black, N = 587 FECs from 11 molecules) shows a major peak and a minor peak as in Fig. 2d. Fitting to the two-population landscape model (red) yields the same relative proportions of N and N′ as for the 6-nt spacer, respectively 89 ± 3% and 11 ± 3%, indicating that the handle has minimal to no effect on the pseudoknot folding when more than 6 nt away.

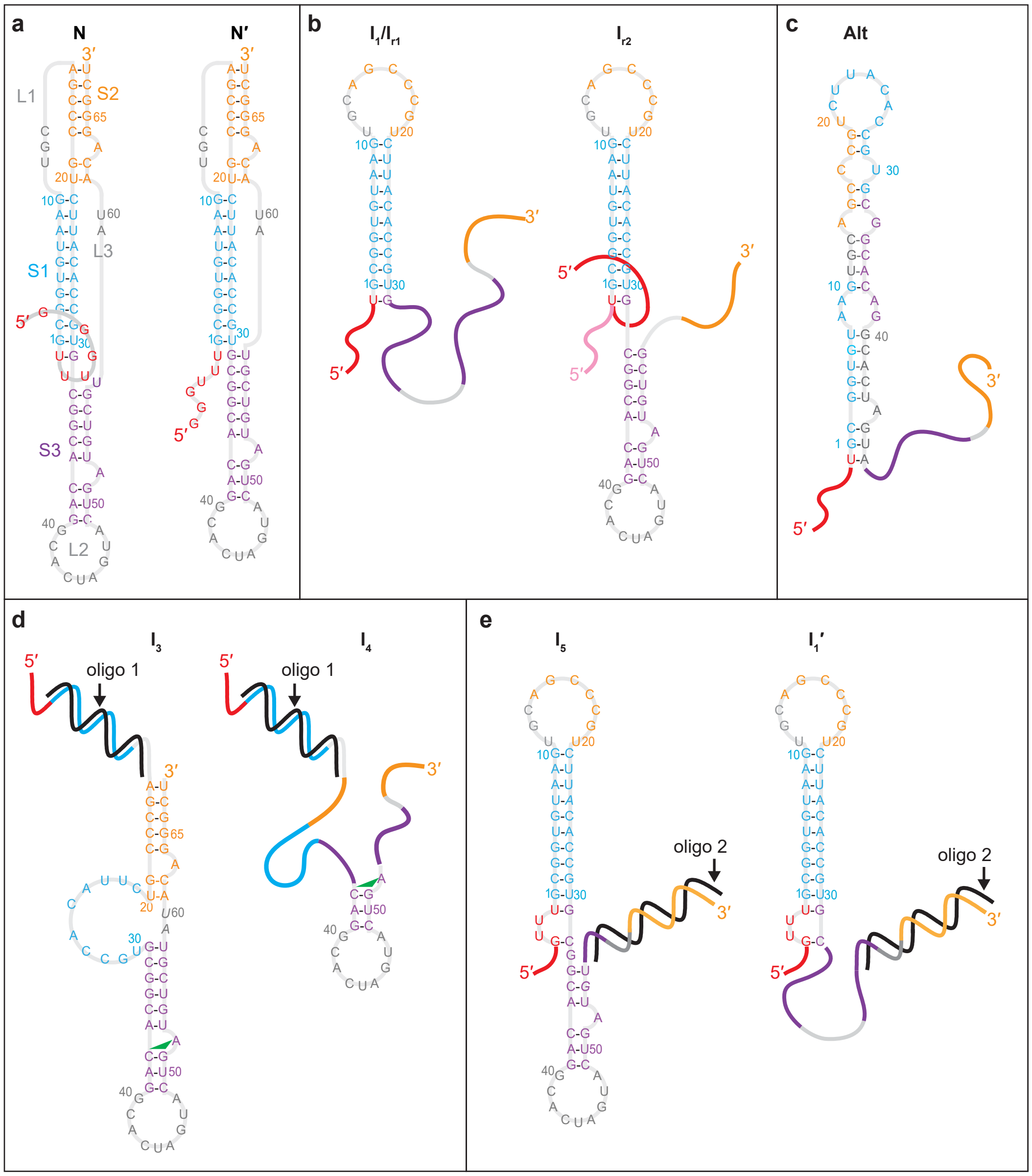

Supplementary Fig. 4: Models of intermediate structures observed in FECs. (**a**) 5′-threaded (left) and unthreaded (right) conformers of pseudoknot, as per models in Ref. 18. (**b**) Unfolding and refolding intermediates with stem 1 only (left) and both stems 1 and 3 (right). For I_{r2}, the geometry of the 5′ end just before closure of stem 2 determines the fold topology: threaded (red) or unthreaded (pink). (**c**) Model of alternative stem-loop structure, which prevents pseudoknot formation. (**d**) Structures formed with oligo 1 bound to stem 1. Intermediate I_4 is proposed to be stabilized by the C37:G51:A52 triple (green triangle) found in Ref. 18. (**e**) Structures formed with oligo 2 bound to stem 2. Stem 1 is extended to pair with some of the unpaired part of stem 3.

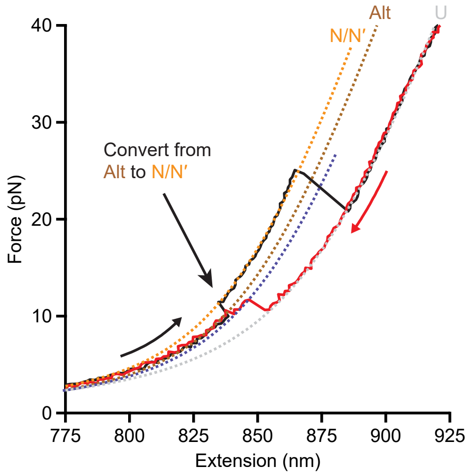

Supplementary Fig. 5: Conversion of Alt to N/N′. An example of a refolding-unfolding cycle in which the RNA first folded into Alt in the refolding FEC (red), and then switched from Alt to N/N′ in the subsequent unfolding FEC (black). Dashed lines: WLC fits.
